# Pulmonary function and physical fitness in obese children and adolescents: a comparison of impulse oscillometry, spirometry, and 6-min walk test

**DOI:** 10.1007/s00431-026-07240-7

**Published:** 2026-07-09

**Authors:** Koravich Na Nakorn, Kanokporn Udomittipong, Puthita Saengpanit, Prakarn Tovichien, Pawinee Charoensittisup, Khunphon Mahoran

**Affiliations:** 1 Division of Pulmonology, Department of Pediatrics, Faculty of Medicine Siriraj Hospital, Mahidol University, 2 Wanglang Road, Bangkok Noi, Bangkok, 10700 Thailand; 2 Division of Nutrition, Department of Pediatrics, Faculty of Medicine Siriraj Hospital, Mahidol University, Bangkok, Thailand

**Keywords:** Impulse oscillometry, Obesity, Pulmonary function, Six-minute walk test, Spirometry

## Abstract

Obesity-associated alterations in pulmonary function have been inconsistently identified by spirometry in pediatric populations. Impulse oscillometry (IOS) offers sensitive detection of peripheral airway resistance, yet data remain limited. Moreover, the relationship between obesity indices and pulmonary function has not been comprehensively evaluated. This study aimed to compare pulmonary function and physical fitness between obese and normal-weight children and adolescents using spirometry, IOS, and the 6-min walk test (6-MWT), and assessed obesity indices as predictors of pulmonary outcomes. We enrolled 120 participants aged 8 to 15 years (obese: *n* = 82; normal weight: *n* = 38). Anthropometry included body mass index (BMI), waist circumference-to-height ratio (WHR), and chest circumference-to-height ratio (CCHR). Pulmonary function was assessed by IOS and spirometry, and physical fitness by the 6-MWT. Obese participants exhibited higher forced vital capacity (FVC) percent predicted (114 ± 17 vs 106 ± 12; *P* = 0.02) and FVC *z*-score (1.09 ± 1.35 vs 0.52 ± 1.00; *P* = 0.02). The ratio of volume expired in 1 s to FVC was reduced (85.0 ± 5.3 vs 88.1 ± 5.8; *P* = 0.005), with no significant change in volume expired in 1 s. The 6-MWT distances were shorter in the obese group (542 ± 45 m vs 583 ± 59 m; *P* < 0.001), and *z*-score-adjusted distances were lower (0.29 ± 0.75 vs 1.24 ± 0.75; *P* < 0.001). Height- and sex-adjusted IOS parameters (R_5_, R_20_, R_5−20_, Fres, AX) were elevated in obesity. Regression analysis identified BMI, WHR, and CCHR as independent predictors of reduced 6-MWT distance and increased R_5−20_.

*Conclusion*: Pediatric obesity is associated with increased total, central, and peripheral airway resistance, and impaired physical fitness. BMI, WHR, and CCHR predict exercise limitation, while WHR and CCHR predict peripheral airway resistance.

**Table:** 

**What is Known:** • *Pediatric obesity is associated with reduced FRC, lower FEV1/FVC ratios, and impaired physical fitness measured by the 6-min walk test.*	
**What is New:** • *Impulse oscillometry (IOS) reveals increased total and peripheral airway resistance in obese children and adolescents, not evident on spirometry.* • *Central adiposity markers, specifically waist circumference-to-height ratio (WHR) and chest circumference-to-height ratio (CCHR), are robust independent predictors of elevated airway resistance and functional limitations.*

**What is Known:**

• *Pediatric obesity is associated with reduced FRC, lower FEV1/FVC ratios, and impaired physical fitness measured by the 6-min walk test.*

**What is New:**

• *Impulse oscillometry (IOS) reveals increased total and peripheral airway resistance in obese children and adolescents, not evident on spirometry.*

• *Central adiposity markers, specifically waist circumference-to-height ratio (WHR) and chest circumference-to-height ratio (CCHR), are robust independent predictors of elevated airway resistance and functional limitations.*

## Introduction

Obesity is a critical global health challenge because its prevalence is increasing among children and adults [1] and it profoundly affects multiple organ systems, including cardiovascular, metabolic, and pulmonary systems [2, 3]. Impaired lung function represents a major pulmonary consequence of obesity [4].

Previous studies and meta-analyses [4–7] reported that obesity in children and adolescents significantly reduces functional residual capacity. It also lowers the ratio of forced expiratory volume in 1 s (FEV_1_) to forced vital capacity (FVC; FEV_1_/FVC), despite normal or elevated FEV_1_ and FVC.

Impulse oscillometry (IOS) is a novel method for assessing respiratory mechanics, and it can detect increased airway resistance in obese children and adults even when spirometry findings appear normal [8–10]. Moreover, IOS demonstrates greater sensitivity than spirometry for resistance in both central and peripheral airways, making it a preferable tool for evaluating small airway function [11]. However, studies comparing lung function measurements obtained by IOS with those obtained by spirometry in children and adolescents with obesity remain sparse and inconclusive.

The 6-min walk test (6-MWT) is a safe, simple, and low-cost method to assess physical fitness, correlating with daily performance [12, 13]. It shows good reproducibility and validity in obese children and adolescents [13]. Therefore, it is well suited to evaluate functional fitness in individuals with obesity who often experience exertional dyspnea. However, studies of 6-MWT in obese pediatric populations remain limited.

Interest in IOS for lung function assessment in obese children is growing. Nevertheless, few studies [14–18] have examined its relationship with obesity-related parameters such as body mass index (BMI), waist circumference, and chest circumference. Even fewer have assessed associations with indices that incorporate height, including waist circumference-to-height ratio (WHR) and chest circumference-to-height ratio (CCHR).

The present study compared lung function in obese children and adolescents using spirometry, IOS, and the 6-MWT. We also examined associations between lung function measures and obesity indices, including BMI, WHR, and CCHR.

Findings may yield insights into respiratory system alterations, particularly airway resistance, in obese pediatric populations. They may also clarify mechanisms underlying exercise intolerance and inform the clinical management of obesity-related respiratory impairment.

## Methods

### Study protocol

We conducted a cross-sectional study from May 2024 to April 2025 at the Department of Pediatrics, Faculty of Medicine Siriraj Hospital, Mahidol University, Bangkok, Thailand. Participants included children and adolescents aged 8 to 15 years with obesity and age- and sex-matched normal-weight controls. Obesity was defined by a BMI *z*-score for age greater than 2 according to World Health Organization growth standards [19], whereas normal weight corresponded to a *z*-score between − 1 and + 1. Exclusion criteria included a respiratory tract infection within 4 weeks before testing, physician-diagnosed asthma, a history of recurrent wheezing, use of asthma control medications, and other chronic respiratory diseases such as bronchiectasis, cystic fibrosis, interstitial lung disease, or bronchopulmonary dysplasia. Participants with cardiovascular, neurological, neuromuscular disorders, skeletal abnormalities affecting lung function, or inability to complete the assessments were also excluded. Participants and guardians provided written informed consent after the research protocol and procedures were explained. We collected anthropometric data before sequential measurements of IOS, spirometry, and 6-MWT, with at least 5 min between each assessment to minimize fatigue effects. The Siriraj Institutional Review Board approved the study (reference: Si 335/2024).

We recorded demographic and obesity-related variables including age, sex, height, body weight, BMI, BMI *z*-score, chest circumference, waist circumference, CCHR, and WHR. All pulmonary assessments were performed by a single trained technician to ensure consistency and reliability.

### Anthropometric data

Anthropometric measurements were obtained using standardized equipment (Tanita Corporation, Tokyo, Japan). We recorded body weight and height to calculate BMI, defined as weight in kilograms divided by height in meters squared. We adjusted BMI for age and sex to derive the *z*-score based on World Health Organization growth standards [20]. We measured chest circumference at nipple level, waist circumference at the midpoint between the lowest rib and iliac crest, and buttocks circumference at the widest part of the buttocks.

### IOS measurement

IOS was performed using a Vyntus IOS device (Vyaire Medical, Mettawa, IL, USA) according to European Respiratory Society guidelines [21]. Participants sat with their head in a neutral position, sealed their lips around the mouthpiece, and wore nose clips to prevent air leakage. An investigator supported each cheek with a palm to minimize pressure loss. Participants breathed normally for at least 16 s per trial, with brief pauses between trials. Trials free of artifacts—such as coughing, vocalization, breath-holding, mouth opening, or swallowing—were repeated until 3 consistent results, each with a coefficient of variation ≤ 15%, were obtained. We calculated the mean of the 3 best trials.

We recorded resistance at 5 Hz (R_5_) and at 20 Hz (R_20_) to represent total and central airway resistance, respectively. We calculated small airway resistance as the difference between these values (R_5−20_). We assessed reactance at 5 Hz (X_5_) to indicate airway restriction, while the area of reactance (AX) and the resonance frequency (Fres) indicated peripheral airway obstruction and lung compliance [10].

### Spirometry

Spirometry was performed using a whole-body plethysmograph (Vyntus BODY; Vyaire Medical, Mettawa, IL, USA) following American Thoracic Society and European Respiratory Society guidelines [22]. We recorded FVC, FEV_1_, the ratio of these values, and forced expiratory flow between 25 and 75% of vital capacity (FEF_25‒75%_). Except for the FEV_1_/FVC ratio, we expressed all values as percent predicted and as *z*-scores based on the 2012 multiethnic global lung function equations [23].

### Six-minute walk test

The 6-MWT, as recommended by the American Thoracic Society [12], assesses functional fitness over a flat, 30-m course marked by a turnaround cone. Participants walked at maximal speed without running for 6 min, with rest breaks as needed. We stopped the test for symptoms such as dyspnea, chest pain, palpitations, or leg cramps. We recorded the total distance in meters and converted it to a *z*-score using Asian pediatric reference equations [24].

### Statistical analysis

Data are presented as means with standard deviations or as counts (%). We compared continuous variables using Student’s *t* test and categorical variables using the chi-square or Fisher’s exact test.

We performed multiple linear regression for each IOS parameter, with group (control vs obese) as the primary predictor and height and sex as covariates, to estimate adjusted mean differences. We also conducted regression analyses to identify which obesity components most strongly influence lung function. Statistical significance was set at *P* < 0.05.

All analyses were conducted in IBM SPSS Statistics version 29 (IBM Corp, Armonk, NY, USA).

## Results

One hundred twenty children participated: 82 (68.3%) in the obese group and 38 (31.7%) in the normal-weight group. Mean age did not differ between groups. The obese group included a higher proportion of males and was significantly taller. All anthropometric measures—body weight, BMI, BMI *z*-score, chest circumference, waist circumference, CCHR, and WHR—were markedly greater in the obese cohort (Table 1).

**Table 1:** Demographic and anthropometric characteristics of control and obese groups

Variable	Mean ± SD		*P* value
	Control group (*n* = 38)	Obese group (*n* = 82)	
Age (years)	11.05 ± 1.87	11.75 ± 1.85	0.06
Male sex, *n* (%)	21 (55.3%)	62 (75.6%)	**0.025**
Height (cm)	143.68 ± 12.34	153.34 ± 12.62	** < 0.001**
Weight (kg)	36.62 ± 8.32	76.06 ± 24.37	** < 0.001**
BMI (kg/m^2^)	17.49 ± 1.58	31.64 ± 6.69	**-**
BMI (*z*-score)	0.003 ± 0.61	3.33 ± 1.02	**-**
Chest circumference (cm)	67.51 ± 6.32	97.96 ± 15.39	** < 0.001**
Waist circumference (cm)	65.64 ± 7.20	99.71 ± 16.47	** < 0.001**
CCHR	0.47 ± 0.03	0.64 ± 0.01	** < 0.001**
WHR	0.46 ± 0.04	0.65 ± 0.08	** < 0.001**

*BMI* body mass index, *CCHR* chest circumference-to-height ratio, *WHR *waist circumference-to-height ratio

Data are mean ± SD or *n* (%)

Comparisons: independent-samples *t* test for continuous variables; chi-square test or Fisher’s exact test for categorical variables

*P* < 0.05; significant values shown in bold

In spirometry, the obese group showed significantly higher FVC expressed as percent predicted and *z*-score (both *P* = 0.02). The FEV_1_/FVC ratio was significantly lower in the obese group. There were no significant differences in FEV_1_ or in FEF_25‒75%_ (Table 2).

**Table 2:** Spirometric measures and 6-min walk distance in control and obese groups

Variable	Control group (*n* = 38)	Obese group (*n* = 82)	Mean difference (95% CI)	*P* value
FEV_1_ (%predicted) (*z*-score)	103.87 ± 11.97 0.33 ± 0.98	108.54 ± 14.56 0.69 ± 1.21	− 4.67 (− 10.03, 0.69) − 0.35 (− 0.8, 0.09)	0.08 0.11
FVC (%predicted) (*z*-score)	106.42 ± 12.38 0.52 ± 1.00	113.6 ± 16.69 1.09 ± 1.35	− 7.18 (− 13.19, − 1.16) − 0.57 (− 1.05, − 0.08)	**0.02** **0.02**
FEV_1_/FVC (%)	88.08 ± 5.84	85.02 ± 5.25	3.06 (0.94, 5.17)	**0.005**
FEF_25–75%_ (%predicted) (*z*-score)	92.11 ± 18.94 − 0.41 ± 0.94	95.0 ± 19.31 − 0.33 ± 1.00	− 2.89 (− 10.35, 4.56) − 0.09 (− 0.47, 0.29)	0.44 0.63
6-MWD (m) (*z*-score)	582.63 ± 59.03 1.24 ± 0.75	541.51 ± 44.88 0.29 ± 0.75	41.12 (19.53, 62.71) 0.95 (0.65, 1.24)	** < 0.001** ** < 0.001**

*6-MWD* 6-min walk distance, *CI* confidence interval, *FEV*_*1*_ forced expiratory volume in 1 s, *FVC* forced vital capacity, *FEV*_*1*_*/FVC* ratio of FEV_1_ and FVC, *FEF*_*25–75%*_ forced expiratory flow at 25% to 75% of FVC

Data are mean ± SD or mean difference (95% CI)

Comparisons: independent-samples t test

*P* < 0.05; significant values shown in bold

In the 6-MWT, children with obesity walked a mean distance of 541.5 m, significantly less than the distance covered by normal-weight peers. This difference remained significant when expressed as a *z*-score (Table 2).

By IOS, small airway resistance (the difference between resistance at 5 Hz and resistance at 20 Hz) was significantly higher in the obese group in both absolute and height- and sex-adjusted analyses. After adjustment, resistance at 5 Hz, resistance at 20 Hz, resonance frequency, and area of reactance all differed significantly, whereas reactance at 5 Hz (X_5_) did not (Table 3).

**Table 3:** Impulse oscillometry parameters in control and obese groups

Variable	Control group (*n* = 38)	Obese group (*n* = 82)	Mean difference (95% CI)	*P* value
**R**_**5**_ Absolute value (kPa/L/s) Adjusted height and sex	0.62 ± 0.13 -	0.66 ± 0.18 -	− 0.04 (− 0.11, 0.02) 0.12 (0.07, 0.17)	0.20 ** < 0.001**
**R**_**20**_ Absolute value (kPa/L/s) Adjusted height and sex	0.47 ± 0.09 -	0.46 ± 0.11 -	0.00 (− 0.04, 0.04) 0.05 (0.02, 0.08)	0.92 **0.004**
**R**_**5–20**_ Absolute value (kPa/L/s) Adjusted height and sex	0.15 ± 0.08 -	0.20 ± 0.10 -	− 0.04 (− 0.07, − 0.03) 0.07 (0.04, 0.11)	**0.02** ** < 0.001**
**X**_**5**_ Absolute value (kPa/L/s) Adjusted height and sex	−0.15 ± 0.05 -	−0.13 ± 0.06 -	− 0.01 (− 0.04, 0.01) − 0.01 (− 0.03, 0.01)	0.16 0.36
**Fres** Absolute value (Hz) Adjusted height and sex	21.47 ± 4.59 -	22.22 ± 4.9 -	− 0.75 (− 2.62, 1.12) 2.63 (0.92, 4.34)	0.42 **0.003**
**AX** Absolute value (kPa/L) Adjusted height and sex	1.39 ± 0.78 -	1.71 ± 1.19 -	− 0.32 (− 0.74, 0.98) 7.75 (3.87, 11.64)	0.13 ** < 0.001**

*R*_*5*_ resistance at 5 Hz, *R*_*5–20*_ difference between R_5_ and R_20_, *R*_*20*_ resistance at 20 Hz, *X*_*5*_ reactance at 5 Hz, *AX* reactance area, *CI* confidence interval, *Fres* resonant frequency

Data are mean ± SD or adjusted mean difference (95% CI)

Comparisons: independent-samples *t* test; adjusted mean differences from multiple linear regression controlling for height and sex

*P* < 0.05; significant values shown in bold

Regression analysis of obesity indices showed that WHR and CCHR predicted increased small airway resistance and reduced 6-MWT distance. BMI and its *z*-score predicted only 6-MWT distance. No significant associations were detected between obesity indices and spirometry parameters (Table 4).

**Table 4:** Associations between obesity indices and pulmonary function and exercise capacity in the obese group (*n* = 82)

Pulmonary function test parameters	Independent variables [1]							
	BMI (kg/m^2^)		BMI (*z*-score)		WHR		CCHR	
	*b*	*P*	*b*	*P*	*b*	*P*	*b*	*P*
R_5_ (kPa/L/s)	0.003	0.19	0.03	0.07	0.35	0.05	0.32	0.11
R_20_ (kPa/L/s)	0.000	0.76	0.01	0.32	0.05	0.65	0.01	0.91
R_5–20_ (kPa/L/s)	0.003	0.08	0.02	0.07	0.30	**0.01**	0.30	**0.02**
X_5_ (kPa/L/s)	− 0.002	0.08	− 0.01	0.09	− 0.13	0.10	− 0.15	0.08
AX (kPa/L)	0.023	0.22	0.12	0.27	2.49	0.08	2.36	0.13
Fres (Hz)	0.009	0.90	0.02	0.07	5.64	0.33	3.19	0.61
FEV_1_ (%predicted)	0.063	0.81	1.00	0.53	− 8.44	0.68	4.53	0.83
FEV_1_ (*z*-score)	− 0.003	0.88	0.03	0.84	− 1.11	0.51	− 0.46	0.80
FVC (%predicted)	0.202	0.51	1.80	0.32	− 9.16	0.69	7.13	0.77
FVC (*z*-score)	0.010	0.68	0.10	0.49	− 1.08	0.56	0.02	0.99
FEV_1_/FVC (%)	− 0.100	0.29	− 0.84	0.13	− 2.73	0.70	− 4.33	0.57
FEF_25–75%_ (%predicted)	− 0.23	0.52	− 1.43	0.51	− 23.59	0.39	− 11.38	0.70
FEF_25–75%_ (*z*-score)	− 0.02	0.25	− 0.14	0.22	− 1.62	0.25	− 1.37	0.37
6-MWD (m)	− 3.72	** < 0.001**	− 19.37	** < 0.001**	− 232.00	** < 0.001**	− 223.93	**0.001**
6-MWD (*z*-score)	− 0.06	** < 0.001**	− 0.31	** < 0.001**	− 3.66	** < 0.001**	− 3.61	**0.001**

*6-MWD* 6-min walk distance, *R*_*5*_ resistance at 5 Hz, *R*_*5–20*_ difference between R_5_ and R_20_, *R*_*20*_ resistance at 20 Hz, *X*_*5*_ reactance at 5 Hz, *AX* reactance area, *BMI* body mass index, *CCHR* chest circumference-to-height ratio, *FEF*_*25–75%*_ forced expiratory flow at 25% to 75% of FVC, *FEV*_*1*_ forced expiratory volume in 1 s, *FEV*_*1*_*/FVC* ratio of FEV_1_ and FVC, *Fres* resonant frequency, *FVC* forced vital capacity, *PEF* peak expiratory flow, *WH*R waist circumference-to-height ratio

Results from multiple linear regression models adjusted for height and sex

Dependent variables: impulse oscillometry parameters, spirometric indices, and 6-MWD (absolute and *z*-score)

Independent variables: BMI, BMI *z*-score, WHR, CCHR

*P* < 0.05; significant coefficients shown in bold

## Discussion

Childhood obesity is a critical global public health issue. Between 1975 and 2016, its global age-standardized prevalence escalated from 0.7% to 5.6% in females and 0.9% to 7.8% in males. This increase contributes significantly to pediatric respiratory morbidity; evidence confirms that excessive body weight independently predicts an increased risk of asthma and respiratory symptoms (relative risk = 1.2–1.8) [25]. Calcaterra et al. [25] stated that obesity-related respiratory dysfunction substantially contributes to chronic pediatric respiratory diseases and may adversely affect health trajectories into adulthood. These findings underscore the need for sensitive tools for the early detecting obesity-related respiratory abnormalities. Our data provide evidence that obesity-related pulmonary alterations can be detected even when conventional spirometric parameters remain largely preserved. Specifically, the increased airway resistance identified by IOS and the reduced functional capacity in the 6-MWT suggest that obesity exerts relevant effects on respiratory function before overt spirometric abnormalities become apparent.

Our study found that the FEV_1_/FVC ratio was significantly lower in obese participants than in normal-weight peers. However, percent-predicted FEV_1_ and FEF_25‒75%_ did not differ between groups and even trended higher in the obese cohort. Thus, a reduced ratio does not indicate obstructive airflow limitation. These results align with previous spirometry studies reporting lower FEV_1_/FVC ratios without obstruction and unchanged or increased FEV_1_ and FVC in obese children and adolescents [4, 6]. This pattern likely reflects dysanapsis, in which disproportionate lung parenchyma growth relative to airway caliber produces greater increases in FVC than in FEV_1_, thereby lowering the ratio [4, 26]. Our observation of higher percent-predicted and *z*-score FVC in the obese group further supports a dysanaptic growth pattern.

Although spirometry did not reveal increased airflow limitation in the obesity group, IOS adjusted for height and sex demonstrated the obese group had higher R_5_, R_20_, R_5–20_, Fres, and AX values, suggesting increased total (R_5_), central (R_20_), and peripheral or small airway resistance (R_5–20_, Fres, AX).

Few studies have compared IOS with spirometry in obese pediatric populations [8, 16, 17]. Our findings agree with prior reports showing elevated airway resistance by oscillometry despite similar spirometric values [8, 16, 17]. Wilhite et al. reported significant increases in total resistance, central resistance, small airway resistance, reactance area, and resonance frequency [17]. Assumpcao et al. [8] reported increased total airway resistance, reactance area, and resonance frequency in children with obesity, despite no significant difference in reactance at low frequencies (X5). Although R5–20, a marker of peripheral airway resistance, was not assessed in their study, the elevated AX and Fres may still suggest increased peripheral airway obstruction, even in the absence of X5 changes [8]. In contrast to our findings and those of Wilhite et al. [17], Assumpção et al. [8] reported no statistically significant difference in central airway resistance (R_20_) between children with and without obesity. Ekstrom et al. observed elevated total resistance, small airway resistance, and reactance area but did not measure central resistance or resonance frequency [16]. All 3 studies, like ours, showed no difference in reactance at 5 Hz [8, 16, 17].

For preschool children, one study reported that obesity increased airway resistance, evidenced by higher R_5_, resonance frequency, and reactance area [14]. Another found elevated total (R_5_) and peripheral airway resistance (R_5−20_, AX) in obese preschoolers [18]. In contrast, Lauhkonen et al. observed no differences in IOS parameters between obese and normal-weight groups. However, they studied preschool children who were hospitalized for acute bronchiolitis between 0 and 6 months old [15]. Similar findings in adults demonstrate increased airway resistance by IOS in obesity despite normal spirometry results [9, 27].

Most IOS studies in pediatric obesity demonstrate increased total and peripheral airway resistance, although central resistance findings are inconsistent. Moreover, IOS detects airway resistance more sensitively than spirometry in both central and peripheral airways, making it the preferred method for small airway evaluation [28, 29].

The mechanisms for increased airway resistance in obese children *are likely multifactorial*. Reduced functional residual capacity from excess abdominal and thoracic adiposity may play a role [30, 31]. Supporting this, one study found that lower functional residual capacity correlated with higher total and peripheral resistance measured by IOS and body plethysmography [17]. Beyond the mechanical effects of excess thoracic and abdominal adiposity, obesity is increasingly recognized as a chronic low-grade inflammatory condition. Adipose tissue produces numerous pro-inflammatory mediators, including leptin, tumor necrosis factor-α, interleukin-6, and other adipokines that may influence airway structure and function. Previous studies have suggested that systemic inflammation may contribute to airway remodeling, altered airway smooth muscle function, and increased airway resistance [15, 17, 25, 27, 32, 33]. Consequently, the elevated increased airway resistance observed in our study may reflect a combination of mechanical and obesity-associated inflammatory processes. Because inflammatory biomarkers were not assessed, we were unable to characterize inflammatory endotypes or determine the contribution of obesity-related inflammation to the increased airway resistance. Future studies integrating lung function testing with systemic and airway inflammatory markers, such as blood eosinophils, fractional exhaled nitric oxide (FeNO), or systemic inflammatory cytokines, may provide greater insight into the pathways linking obesity and pulmonary dysfunction.

Bronchodilator test was not performed in our study because the study focused on baseline pulmonary function in children without known respiratory disease. Participants with physician-diagnosed asthma and other chronic pulmonary disorders were excluded to minimize confounding from established airway disease. However, evaluating the reversibility of bronchial obstruction is fundamental to a comprehensive lung function test and could provide additional insight into whether the elevated airway resistance detected by IOS reflects reversible bronchial dysfunction or predominantly obesity-related mechanical alterations [34]. Further studies should consider integrating bronchodilator test to differentiate between structural, obesity-induced mechanics and reversible airway hyperresponsiveness.

In our study, obese children walked shorter distances in the 6-MWT, both in absolute meters and *z*-scores, compared to normal-weight controls. This finding aligns with previous research that demonstrated the detrimental effects of obesity on functional capacity [7, 35–37]. Excessive adiposity impairs lung mechanics and chest wall compliance [35, 38], increases energy demand, and raises the mechanical cost of movement [39], thus reducing functional exercise capacity and contributing to exertional dyspnea. These results suggest that IOS and the 6-MWT are more sensitive than spirometry for detecting obesity-related pulmonary impairment in pediatric populations.

Associations between BMI, WHR, and CCHR and pulmonary function were assessed using IOS, spirometry, and the 6-MWT. WHR and CCHR emerged as strong predictors of increased small airway resistance (R_5−20_) and reduced 6-MWT distance. BMI and its *z*-score predicted only 6-MWT distance. No significant associations were detected between any obesity index and spirometry parameters.

Few studies have examined 6-MWT distance in obese children and adolescents. Most investigations evaluated BMI or its *z*-score, reporting significant correlations with walk distance [13, 35, 40]. Two studies that also included WHR alongside other indices—such as BMI or its *z*-score, chest circumference, and waist circumference—found its strong correlations with 6-MWT distance [7, 41]. Small airway resistance (R_5−20_) correlated positively with WHR and CCHR but showed no relationship with BMI in our cohort. No prior studies in obese children have examined IOS associations with these height-adjusted indices. Ekstrom et al. [16] and Wilhite et al. [17] reported R_5−20_ associations with BMI but did not assess WHR or CCHR. In preschoolers aged 3‒7 years, one study found R_5_ and R_5−20_ correlated with WHR [18]. Lauhkonen et al. [14] observed BMI effects on R_5_ but did not report R_5−20_, and Kalhoff et al. found no BMI associations with R_5_ or X_5_ [14]. None of these studies evaluated height-adjusted circumference measures. We included WHR and CCHR because absolute waist and chest circumferences increase with both adiposity and somatic growth, potentially confounding their relationships with airway resistance.

### Limitations

This study has limitations. First, its cross-sectional design precludes causal inference between obesity and pulmonary dysfunction. Second, single-center data may limit generalizability, and multicenter studies with larger samples are necessary. Third, adiposity was not assessed by direct methods such as bioelectrical impedance analysis or dual-energy X-ray absorptiometry, because we prioritized simple, practical measures feasible in routine clinical practice. Fourth, bronchodilator responsiveness testing was not performed. Consequently, we could not determine whether the increased airway resistance observed by IOS represented reversible bronchial obstruction or fixed obesity-related alterations in respiratory mechanics. Finally, we did not evaluate physical activity, diet, or inflammatory status; this lacks the ability to correlate airway resistance with biological inflammatory profiles. Future research should incorporate these variables for a comprehensive understanding of obesity-pulmonary interactions.

## Conclusions

Children and adolescents with obesity showed higher total, central, and peripheral airway resistance measured by IOS. They also walked shorter distances in the 6-MWT. Spirometry revealed a lower FEV_1_/FVC ratio with elevated FVC and a trend toward increased FEV_1_, consistent with dysanapsis. Regression analysis identified BMI, WHR, and CCHR as independent predictors of reduced physical fitness, while WHR and CCHR predicted increased airway resistance. These findings underscore the importance of early weight-management interventions and routine anthropometric monitoring to detect obesity-related pulmonary alterations.

## Data Availability

No datasets were generated or analysed during the current study.
